# Sexuality in Adult Cancer Patients Living with Enterostomy or Urostomy: A Descriptive Phenomenological Study

**DOI:** 10.3390/curroncol33050270

**Published:** 2026-05-07

**Authors:** Nicolò Panattoni, Giulia Manzon, Alessia Campoli, Valentina Anselmi, Francesca Laurenza, Chiara Giammaria, Aurora De Leo, Alessandro Spano, Fabrizio Petrone, Emanuele Di Simone, Laura Iacorossi

**Affiliations:** 1 Nursing Research Unit IFO, IRCCS Regina Elena National Cancer Institute, 00144 Rome, Italy; nicolo.panattoni@ifo.it (N.P.); giulia.manzon@ifo.it (G.M.); alessia.campoli@ifo.it (A.C.); chiara.giammaria@ifo.it (C.G.); aurora.deleo@ifo.it (A.D.L.); fabrizio.petrone@ifo.it (F.P.); 2 Department of Biomedicine and Prevention, University of Rome Tor Vergata, 00133 Rome, Italy; valentina.anselmi@ifo.it (V.A.); francesca.laurenza@ifo.it (F.L.); 3 Department of Medical, Movement and Wellbeing Sciences, Parthenope University of Naples, 80133 Naples, Italy; emanuele.disimone@uniparthenope.it; 4 Department of Life, Health and Health Professions Sciences, Link Campus University, 00165 Rome, Italy; l.iacorossi@unilink.it

**Keywords:** sexuality, cancer, enterostomy, urostomy, phenomenology, nursing, quality of life

## Abstract

Cancer patients with an enterostomy or urostomy face significant physical and psychological challenges that impact their sexuality and quality of life. Despite its importance, this topic is often overlooked in clinical settings. This study explores the lived experiences of these patients regarding their sexual health. Using a descriptive phenomenological approach, researchers performed face-to-face interviews with 33 adult cancer patients living with enterostomy or urostomy at Istituto di Ricovero e Cura a Carattere Scientifico (IRCCS) Regina Elena National Cancer Institute of Rome, Italy. Data were analyzed according to Giorgi’s method to identify core themes. Four primary themes emerged: the emotional weight of surgery, fear of rejection or disgust, the influence of partner reactions on intimacy, and the struggle to find professional guidance. Participants reported reduced desire and altered body image, though many utilized coping strategies like personal resilience and partner support. Stoma surgery profoundly affects sexuality, yet professional support remains inadequate. The study highlights a critical need for multidisciplinary care and proactive communication. Integrating sexual health into routine oncological practice is essential for providing person-centred care and improving overall quality of life.

## Simple Summary

Sexuality is a central aspect of being human throughout life, encompassing sex, gender identities, sexual orientation, eroticism, pleasure, intimacy, and reproduction. For cancer patients with an enterostomy or urostomy, these dimensions undergo profound transformations that significantly impair their sexuality and quality of life . While these challenges are well-documented, sexual health remains a marginalized topic in clinical practice, often excluded from routine oncological care and patient–provider discussions. Utilizing a phenomenological approach, this study explores how patients struggle with altered body image, fear of rejection, and functional limitations. A critical finding is the discrepancy between the patients’ need for sexual rehabilitation and the lack of professional guidance. While resilience and partner support act as vital coping mechanisms, they cannot substitute for structured clinical intervention. These findings support a shift in policy toward person-centered care. By integrating sexual health into multidisciplinary protocols, future studies can develop targeted interventions to mitigate psychological distress. Implementing these insights will foster proactive communication, ultimately improving long-term outcomes and quality of life for stoma patients.

## 1. Introduction

Colorectal cancer is one of the most common cancers worldwide, with the highest incidence rates observed in Australia, New Zealand, and Europe [1]. Bladder cancer is the seventh most commonly diagnosed cancer among men globally [2]. Surgical procedures such as urostomy or enterostomy are often performed to treat bowel or urinary diseases [3]. Although these interventions are frequently lifesaving, the creation of a stoma deeply alters patients’ bodies and daily lives, affecting body image and health-related quality of life [4]. According to the World Health Organization (WHO), sexuality is a central aspect of being human throughout life, encompassing sex, gender identities, sexual orientation, eroticism, pleasure, intimacy, and reproduction. For cancer patients with an enterostomy or urostomy, these dimensions undergo profound transformations that significantly impair their sexuality and quality of life [5]. Sexuality is a crucial component of human well-being [6], yet stoma patients often report significant limitations in their sexual lives and difficulties related to the physical and psychological changes following surgery [7,8]. Recent evidence emphasizes that stoma formation induces profound body image distortions, often characterized by a loss of bodily trust and a perceived sense of ‘mutilation’ that transcends the physical presence of the stoma [9,10]. This altered body image acts as a primary catalyst for sexual dysfunction; patients frequently report feeling ‘asexualized’ or ‘unattractive,’ leading to a significant decline in sexual self-esteem [9,10]. Meta-analytic findings confirm that ostomy patients experience significantly higher levels of body image distress and sexual dissatisfaction compared to non-stomized surgical patients [10], with over 60% of patients reporting unmet needs in this domain [11]. These alterations foster pervasive feelings of isolation, shame, and a persistent fear of rejection or device leakage during intimacy [12]. Despite its impact, sexuality remains a marginalized topic in clinical practice, often obscured by cultural taboos—particularly among older adults—and a lack of proactive professional guidance [13,14,15]. Existing literature highlights a range of sexual difficulties among ostomy patients, including decreased desire and libido [16], impaired arousal [17], anorgasmia [18], erectile dysfunction and ejaculation disorders in men [9], and dyspareunia and vaginal dryness in women [19]. Partner closeness has been identified as a protective factor that supports acceptance of the new body image and facilitates intimacy [20]. However, gaps remain in understanding the specific sexual needs of cancer patients with stomas and in developing tailored educational interventions [9,16]. This study aims to explore the lived experience of sexuality in adult oncology patients with an enterostomy or urostomy, and to identify the factors influencing their sexual and relational well-being, to inform the development of targeted and personalized educational strategies.

## 2. Materials and Methods

### 2.1. Study Design

A descriptive phenomenological design, grounded in Husserl’s philosophical principles [21], was adopted to explore the lived experience of sexuality in adult cancer patients with enterostomy or urostomy [22]. This approach aimed to explore and describe participants’ perceptions, emotions, and meanings without imposing the researchers’ interpretations. Following Husserl’s method, the researchers applied epoché (bracketing) to set aside their own preconceptions and biases, thereby allowing the ‘essence’ of the participants’ lived reality to emerge. This ensured that the analysis remained focused on the pure phenomena of the patients’ experiences, facilitating a rigorous description of the structures of consciousness related to their sexual health and body image [21]. Open-ended, face-to-face, non-judgmental interviews allowed participants to share their experiences freely, generating rich and nuanced descriptions that supported a deep understanding of their lived world [23]. The study followed the COREQ (Consolidated Criteria for Reporting Qualitative Research) guidelines to ensure methodological rigor, transparency, and consistency (Table S1) [24].

### 2.2. Participants

A sample of adult cancer patients living with an enterostomy and/or urostomy was recruited after providing written informed consent. Participation was voluntary, and individuals were informed that they could withdraw from the study at any time, without any modification to the standard care pathway. Potential participants were identified through a review of medical records and in collaboration with the nursing staff, who confirmed eligibility based on the inclusion criteria. Purposive sampling was used to recruit information-rich cases, following Sandelowski’s principles of adequacy and variation [25]. Inclusion criteria required individuals to be ≥18 years old, have a cancer diagnosis, experience no difficulties in verbal communication, and have an enterostomy and/or urostomy. Data collection continued until data saturation was reached [26], meaning that no new relevant information or themes emerged from subsequent interviews, in accordance with Guest, Namey, and Chen’s operational definition [26,27]. Consistent with qualitative descriptive phenomenological methodology, sample adequacy was guided by thematic saturation rather than statistical considerations of representativeness. Saturation was monitored throughout data collection by the research team, who assessed the emergence of new themes after each interview. Participants were recruited from two outpatient units—the Stoma Care Clinic and the Urology Clinic—at the IRCCS Regina Elena National Cancer Institute in Rome, Italy. These two clinical settings were selected because they ensured easy access to patients with different types of stoma and provided continuity of care across the postoperative pathway. Their organizational characteristics allowed the research team to recruit information-rich cases and to capture a wide range of lived experiences. All data were pseudonymized to ensure confidentiality. 

### 2.3. Setting and Data Collection

Data were collected through open-ended interviews conducted by a nurse researcher in a dedicated, quiet, and private room within the Stoma Care Clinic, ensuring an environment conducive to focused and in-depth dialogue. Using one interviewer minimized variability [28]. Interviews took place between April and May 2025. Before each interview, the aims of the study and the interview procedure were clearly explained, and participants were encouraged to speak freely and spontaneously. The interview guide, developed from clinical experience and literature, included four open questions (Table 1).

The guide was pilot-tested with two participants and required no modifications, confirming the clarity and effectiveness of the questions. Each interview lasted approximately 20 to 30 min and was audio-recorded to ensure accurate transcription and analysis.

### 2.4. Data Analysis

The analytic process followed Giorgi’s descriptive phenomenological method, which consists of five sequential steps [29], and was integrated with an inductive content analysis approach [30]. These steps include: (1) collection of verbal data, (2) open and repeated reading to gain a holistic understanding, (3) identification of units of meaning, (4) transformation and organization of these units from a phenomenological perspective, and (5) synthesis into essential structure. In this study, interviews were audio-recorded and transcribed verbatim. Each participant completed one interview; no repeat interviews were conducted. The transcripts were then read repeatedly to grasp a holistic sense of the material. “Units of meaning” were identified, reorganized, and transformed through a phenomenological attitude. Finally, these units were synthesized into categories and themes, capturing the essential structure of the phenomenon. The resulting themes and categories were used to articulate participants’ lived experiences. A third researcher with expertise in qualitative research supervised all analytic phases and resolved discrepancies through discussion until full agreement was reached. No qualitative software was used; instead, grids and tables supported the organization of the data. Inductive content analysis was applied only to support data organization and presentation, while the analytic process remained fully grounded in Giorgi’s descriptive phenomenological method.

### 2.5. Trustworthiness

Trustworthiness was ensured according to Lincoln and Guba’s criteria [28]. Credibility was established through data saturation, researcher triangulation, and consensus discussions. Dependability was strengthened by maintaining procedural consistency and providing a transparent description of all analytic steps. Confirmability was enhanced through verbatim transcription, an attitude free from preconceptions, and supervision by a third researcher [28]. To further reduce interpretive bias, the researchers engaged in bracketing (epoché), intentionally setting aside personal assumptions, clinical experience, and theoretical expectations throughout data collection and analysis. Transferability was facilitated through a detailed description of the study context, participants, and methodological procedures.

### 2.6. Ethical Considerations

The study protocol was reviewed and approved by the Territorial Ethics Committee Lazio Area 5 of the IRCCS Regina Elena National Cancer Institute—IFO (Protocol Code: 13702, 10 October 2024; Experimental Registry No: 224/IRE/24). The research was conducted in strict accordance with the ethical principles outlined in the Declaration of Helsinki [31]. Prior to enrollment, all participants provided written informed consent and were explicitly informed about the voluntary nature of their participation and the absence of financial incentives. To protect subject confidentiality, all data were anonymized during the transcription and analysis phases; the robust de-identification procedures ensured that individual participants could not be retrospectively identified by the research team.

## 3. Results

### 3.1. Socio-Demographic and Clinical Participant Characteristics

A consecutive sample of 33 cancer patients living with an enterostomy and/or urostomy was enrolled in the study, and all participants completed the interview without any losses during the data collection. The sample was predominantly composed of men (*n* = 27; 81.8%), with a mean age of 62 years (range 37–79). Bladder cancer emerged as the most prevalent diagnosis (*n* = 17; 51.5%), followed by rectal cancer (*n* = 7; 21.2%), colon cancer (*n* = 4; 12.1%), and other diseases (*n* = 6; 18.2%). Twenty-four participants had previously undergone chemotherapy (*n* = 24; 72.7%), and ten participants had previously undergone radiotherapy (*n* = 10; 30.3%). The most common type of stoma among the 33 patients was urostomy (*n* = 17; 51.6%), followed by colostomy (*n* = 11; 33.3%) and ileostomy (*n* = 5; 15.1%). Table 2 provides a detailed overview of the demographic characteristics collected. Participants’ time since diagnosis distribution is also presented in Table 2 to reflect the heterogeneity of clinical trajectories.

**Table 2:** Participants’ demographic characteristics.

Patient Characteristic	N (%)
**Age (years), mean ± SD (range)**	62 ± 11.0 (37–79)
<65 years	18 (54.5)
≥65 years	15 (45.5)
**Gender**	
Male	27 (81.8)
Female	6 (18.2)
**Cancer type**	
Colon	3 (9.1)
Rectal	7 (21.2)
Bladder	17 (51.5)
Other	6 (18.2)
**Chemotherapy**	
Yes	24 (72.7)
No	9 (27.3)
**Radiotherapy**	
Yes	10 (30.3)
No	23 (69.7)
**Ostomy**	
Colostomy	11 (33.3)
Ileostomy	5 (15.1)
Urostomy	17 (51.6)
**Time of diagnosis**	
≤6 months	2 (6.1)
>6 months and ≤1 year	7 (21.2)
>1 year and ≤3 years	8 (24.2)
>3 years and ≤5 years	9 (27.3)
>5 years	7 (21.2)

### 3.2. Phenomenological Findings

The phenomenological analysis [26] yielded four themes and ten categories. An illustrative example of the coding process is presented below (Table 3).

The themes identified provide a comprehensive understanding of how individuals living with a colostomy, ileostomy, or urostomy experience sexuality after surgery. Table 4 offers a prevalence overview of the four main themes across participants [32], illustrating the variability and depth of these experiences within the sample.

The analysis shows that sexuality is shaped primarily by emotional and relational factors, while physical changes play a secondary but still relevant role. This thematic structure highlights how participants navigate the complex process of reconstructing their sense of identity and well-being following stoma formation. The four themes reflect distinct yet interconnected dimensions of the participants’ experiences: (1) the emotional impact of sexuality after stoma surgery, (2) the fear of disgust and sexual rejection, (3) partner reactions as a determinant of intimacy, and (4) searching for guidance and support. Together, these themes clarify how sexuality is influenced not only by bodily changes but also by psychological meanings, relational dynamics, and the availability or absence of adequate support. This structure also allows for a nuanced comparison across different types of stomas represented in the sample. Each participant was assigned an alphanumeric code (P1–P33) indicating gender (M = male, F = female), age, and type of stoma (e.g., P1, M, 58 years old, colostomy). The following sections present the four themes and their sub-themes, supported by illustrative quotations from the interviews.

#### 3.2.1. The Emotional Impact of Sexuality After Stoma Surgery

Sexuality after enterostomy or urostomy was experienced by participants as a significant source of distress, involving the body, emotions, and the relational sphere. This distress was multidimensional, arising from the profound transformation of the body and the difficulty in regaining spontaneity and intimacy. Alongside these challenges, some patients demonstrated a notable capacity for acceptance, attributing value to survival and the continuity of life.

##### Physical Distress

Many participants reported physical difficulties that directly interfered with their ability to engage in sexual activity. Erectile dysfunction, reduced stimulation, and loss of energy were among the most frequently mentioned issues:

“*Our sexuality is almost zero. No, it’s… it’s zero. […] I no longer have erections… My desire has also decreased a lot.*”

(P18, M, 79 years, urostomy)

“*Since I have the stoma […] I no longer have that energy and desire*.”

(P9, M, 62 years, ileostomy)

“*Since I had the surgery, I no longer have any sexual activity. I’ve completely lost the drive, the pleasure*.”

(P13, M, 70 years, colostomy)

“*My sexual life ended the day before the surgery*.”

(P15, F, 60 years, colostomy)

In some cases, the anatomical transformation was experienced as an irreversible loss:

“*My sexuality is dead. […] Now it’s just a memory*.”

(P22, M, 75 years, urostomy)

##### Psychological Distress

Several participants described deep psychological distress, characterized by emotional blocks, sadness, and difficulty recognizing themselves in their sexual identity:

“*Sex has disappeared. Since I had these surgeries, I have a block, a psychological block*.”

(P5, M, 70 years, colostomy)

“*Even psychologically, it has changed my sexual relationship with the opposite sex a little bit. This is something I have to be honest about. But it’s a personal thing [of mine]*.”

(P9, M, 62 years, ileostomy)

Some spoke of a sense of “dimming,” as if the stoma had taken away spontaneity and vitality:

“*It has dampened me a bit. Mentally, I’m positive, but this weighs on me*.”

(P11, M, 54 years, ileostomy)

##### Relational Distress

Distress also affected the perception of the relationship. Some participants described a sense of distance, embarrassment, or difficulty letting go as part of their emotional experience after the stoma:

“*Before this happened to me, I had a spirit for going with women; whenever I found [one], whenever I saw [one], I had more spirit. Then, since this thing happened to me—it happened that I had my rectum and everything removed—and now I have withdrawn into myself a bit, and I don’t have that drive anymore*.”

(P6, M, 70 years, colostomy)

“*There are only some intimate attitudes […], but it’s not like before anymore*.”

(P13, M, 70 years, colostomy)

These feelings reflect the patient’s subjective perception, not the partner’s behavior.

##### Acceptance and Adaptation

Alongside distress, some participants expressed a form of acceptance, not to be construed as a “facilitating factor”, but rather as a way of making sense of their experience.

“*It saved my life, and those who accept me, fine, and those who don’t, so be it, so I’ve reached the point where I accept it*.”

(P10, M, 46 years, colostomy)

For these individuals, survival and the continuity of life were the priority, and sexuality was reinterpreted through a personal philosophy that emphasized the value of being alive:

“*You need philosophy. I saved my skin, and that’s what matters*.”

(P25, M, 72 years, urostomy)

“*It’s better to have the stoma than not be here anymore. That’s my philosophy*.”

(P17, M, 63 years, urostomy)

Others highlighted traits of resilience, such as patience, strength of character, and the ability to maintain a positive attitude:

“*I always laugh. Mentally, I’m very positive*.”

(P11, M, 54 years, ileostomy)

#### 3.2.2. The Fear of Disgust and Sexual Rejection

Fear of disgust emerged as the strongest and most pervasive emotional theme. Many participants feared that the stoma might provoke repulsion in their partner, generating insecurity, avoidance of intimacy, and difficulty letting go.

“*The main fear is that it might disgust my partner, because living with a bag of feces on your belly makes you think of something disgusting… and it makes me think of myself that way*.”

(P4, F, 51 years, colostomy)

“*I tried to have intercourse again, but he kept telling me to cover the bag, he felt uncomfortable*.”

(P2, F, 42 years, colostomy)

This fear was intertwined with other emotional dimensions that amplified the distress.

##### Fear of Rejection

Patients fear perceiving rejection from others, even through subtle gestures and/or facial expressions.

“*My fear is being able to read a moment of disgust or rejection in the other person’s gaze, and that is a fear that holds me back*.”

(P4, F, 51 years, colostomy)

##### Compromised Body Image

Findings highlight that patients encounter substantial difficulties adapting to their new physical configuration following stoma placement. This struggle with a ‘new body’ often manifests as a sense of estrangement and diminished self-esteem, emerging as a critical challenge in the postoperative rehabilitation process.

“*Living with a bag of feces on your belly makes you think of something disgusting… and it makes me think of myself that way*.”

(P4, F, 51 years, colostomy)

“*Show them [partners] this body of mine creates an even greater obstacle for me*.”

(P4, F, 51 years, colostomy)

##### Shame

Shame was a recurring and central emotion, leading patients to hide the stoma, avoid nudity, and limit sexual initiative:

“*I always try to hide it… But whether I want to or not, it shows, and that makes me ashamed*.”

(P11, M, 54 years, ileostomy)

“*If this smell is also felt by the partner, it is truly a shame, because […] this is the smell of feces, and this is not a smell, this is a stench. And this is truly a great embarrassment and shame*.”

(P15, F, 60 years, colostomy)

Shame was found to be more prevalent in patients without a stable partner, as some patients with a stable partner stated they did not experience this emotion due to their emotional bond with the other.

“*I’m ashamed, if you’re ashamed […] Why should you be ashamed in front of your partner*?”

(P7, M, 50 years, colostomy)

##### Difficulty Showing Oneself and Feeling Desirable

Qualitative evidence suggests that patients experience significant distress regarding physical nudity, primarily due to concerns over odors, the pouching system, and potential leakage. These factors fragment body image and diminish perceived desirability, creating psychological barriers to intimacy. Furthermore, for these individuals, the concept of ‘beauty’ remains fundamentally difficult to reconcile with the reality of a stoma.

“*The concept of beautiful and stoma is something that seems almost like an oxymoron, because the first time, I cried so much out of disgust that it took me a long time to learn to manage it alone, because I rejected it (teary eyes) […] I don’t like it, I tried to give it a nickname [to stoma], but we’re still not on good terms*.”

(P4, F, 51 years, colostomy)

“*The problem is mainly mine, how I see myself, how much confidence I can have right now, even with a stoma*.”

(P4, F, 51 years, colostomy)

“*I also encountered difficulties with some women who simply wouldn’t accept this stoma, the pouch, and everything else*.”

(P10, M, 46 years, colostomy)

These dimensions contributed to a sense of fragility that limited sexual initiative and made the resumption of intimacy complex.

#### 3.2.3. Partner Reactions as a Determinant of Intimacy

Participants described how the partner’s reactions played a decisive role in shaping their sexual experiences after stoma surgery. These reactions could either support the resumption of intimacy or generate discomfort, influencing how participants approached sexual activity.

##### Supportive Partner Reactions

Several participants described partners who responded with acceptance, reassurance, and emotional closeness. These reactions helped reduce guilt, contain the fear of disgust, and foster a sense of safety during intimate moments. Supportive partners were perceived as actively contributing to the reconstruction of intimacy:

“*The stoma didn’t bother my partner; on the contrary, she was welcoming about it, and this most likely allowed me not to experience it as disabling*.”

(P12, M, 60 years, ileostomy)

“*My wife was the first to accept this new situation, even before I did*.”

(P17, M, 63 years, urostomy)

“*My wife helped me a lot, even psychologically, to avoid making me feel guilty*.”

(P26, M, 65 years, urostomy)

“*My partner told me: ‘Nothing has changed for me. I’m fine with you anyway.’ That helped me a lot*.”

(P24, M, 79 years, urostomy)

“*When you have someone who truly cares about you and loves you for who you are, not for what you look like […] they accept everything, also because […] there are worse things, […] you are a normal person just like everyone else*.”

(P3, F, 51 years, colostomy)

##### Partner Reactions That Created Discomfort

Other participants reported partner reactions that generated unease during intimacy. Expressions of discomfort, hesitation, or difficulty in approaching the stoma were experienced as emotionally challenging and contributed to increased insecurity:

“*Those reactions […] from my wife during the first [pouch] changes, they were reactions that were certainly not along the lines of “look how beautiful it is”… I mean, I saw certain types of expressions in her too; so, those expressions […] hit you*.”

(P7, M, 50 years, colostomy)

“*I tried to have intercourse, but he told me to cover the bag because it made him uncomfortable*.”

(P2, F, 42 years, colostomy)

“*She sees it, so it bothers her to see it*.”

(P11, M, 54 years, ileostomy)

Across accounts, partner reactions—whether supportive or discomforting—emerged as a central factor influencing participants’ willingness to engage in intimacy after stoma surgery.

#### 3.2.4. Searching for Guidance and Support

A transversal theme concerned the lack of information and psychological support received before and after surgery. Participants expressed a clear need to be guided in understanding the bodily, emotional, and relational changes associated with the stoma, particularly regarding sexuality. Many reported feeling unprepared to face the consequences of surgery on their intimate life, describing an informational void that amplified anxiety, uncertainty, and loneliness:

“*I wasn’t told anything about what I would have to face. A minimum of information would have been essential*.”

(P20, M, 73 years, urostomy)

“*I didn’t receive any information at all*.”

(P21, F, 65 years, urostomy)

Beyond clear information, participants expressed the need for ongoing patient education, helping them understand what to expect and how to manage changes in daily and intimate life:

“*I found out through […] Google and all that […] that by removing those little nerves, the ones for sexuality […] you would remain impotent in this way*.”

(P19, M, 57 years, urostomy)

“*Perhaps someone could have helped me or explained that it’s normal*.”

(P21, F, 65 years, urostomy)

“*I was simply told beforehand: ‘There will be a decrease in sexuality’. Some ‘decrease’—we’re at zero. Well, maybe this would have prepared me more*.”

(P22, M, 75 years, urostomy)

“*A little more attention, especially when you are brought back to the room; at least one doctor showing up […] maybe introducing themselves to the patient and saying: ‘Look, we did this and we’re moving forward.’ Instead, unfortunately, I didn’t receive this attention*.”

(P32, M, 60 years, urostomy)

Many emphasized the need for structured psychological support, addressing not only stoma management but also fear of recurrence, loss of spontaneity, and changes in self-perception:

“*In my opinion, psychological support must exist because accepting yourself is not simple. I understand that it is not simple. Psychological support could be useful for many things*.”

(P14, F, 45 years, colostomy)

Others highlighted the potential value of support groups, perceived as spaces to share fears, doubts, and common experiences:

“*Maybe if there was a group to go to, they could have told me, to participate in a group or something that would help me […] or told me, for example, that it’s normal*.”

(P21, F, 65 years, urostomy)

Finally, participants expressed the need to address sexuality both before and after surgery, without the topic being avoided or minimized:

“*The patient should be put in a position to start a journey […] right from the start, to guide them toward regaining a bit of sexual serenity as well*.”

(P17, M, 63 years, urostomy)

“*I would suggest to those in charge of these things to talk to patients a bit; I was hospitalized for six days and after the surgery someone could have […] told me, to join a group or something that would help me from this point of view, or that would tell me, for example, that I wouldn’t have the desire. I, who never had these problems, I always made love with my husband all the time, a lot, often, and then suddenly my desire just went away*.”

(P21, F, 65 years, urostomy)

Within this process, the nurse was perceived as the most proximate and approachable professional, able to identify distress early on, provide initial emotional support, deliver clear and personalized information, and refer patients to specialists or support groups when necessary.

## 4. Discussion

This study explored how individuals with an enterostomy or urostomy experience sexuality after surgery. The findings reveal a complex interplay of physical, psychological, relational, and informational dimensions. Consistent with existing literature, sexuality emerged as a deeply embodied and relational experience, rather than a marginal aspect of life after stoma formation. Participants reported significant physical and psychological challenges that interfered with sexual functioning, echoing earlier studies documenting reduced sexual desire, erectile dysfunction, fatigue, and altered bodily sensations among stoma patients [33,34,35]. These findings align with research on sexual dysfunction following colorectal cancer treatment [19,20,36]. Our study adds a deeper phenomenological perspective, showing how these changes are experienced as a loss of vitality and identity, consistent with interpretative analyses of living with an altered body [37,38]. Some participants expressed an existential acceptance grounded in survival, a reframing also described by Charmaz (1995) and, more recently, observed in qualitative studies on marital intimacy after ostomy creation [16,39]. Difficulties in accepting the altered body, shaped by the pouch, perceived odors, and bodily changes, were also evident [9,20,40]. Fear of disgust emerged as the most pervasive emotional theme, consistent with literature on altered body image among cancer and stoma patients [8,41,42]. Our findings deepen this understanding by showing how disgust is often internalized. Participants frequently described perceiving themselves as “disgusting”, reinforcing a negative self-image and contributing to sexual avoidance [9]. Shame, fear of rejection, and difficulty feeling desirable were closely intertwined with this fear of disgust. Together, these emotions contributed to a self-reinforcing cycle of sexual avoidance. Similar patterns have been reported in previous qualitative studies on sexuality after stoma formation [43,44] and among female colorectal cancer survivors [45]. Contrary to the notion that low self-disclosure and absent communication invariably lead to marital strain, we found that patients in stable relationships receive significant support and understanding, which serves as a crucial factor in accepting their new condition. The communication barriers highlighted in earlier literature appear more prevalent among patients without a stable partner [46,47,48]. The findings confirm that sexuality after stoma surgery is inherently dyadic. Partner reactions and behaviors strongly shaped participants’ experiences of intimacy. This aligns with previous studies showing that partner support plays a crucial role in adjustment and quality of life [49,50,51]. Supportive partners helped participants feel accepted and reduced guilt, whereas partners who expressed discomfort intensified insecurity and relational strain, as also observed in Chinese and international samples [16,52]. These findings are consistent with relational theories of chronic illness, which conceptualize adaptation as a co-constructed process within the couple [53]. Participants consistently reported a lack of information and psychological support regarding sexuality. This confirms a gap widely documented in the literature [54,55,56]. Healthcare professionals rarely initiate conversations about sexual health with stoma patients. As a result, many participants sought information through alternative sources, such as the internet or social media [57]. Our study confirms this trend. Most participants reported a lack of adequate information after surgery. Many did not raise the topic of sexuality with clinicians and instead searched for answers independently. Their needs extended beyond technical guidance on stoma management to include clear, honest, and anticipatory information about how their intimate life might change, as highlighted by Black (2004) and reinforced by recent reviews [7,58]. The need for ongoing patient education and structured psychological support is consistent with evidence showing that educational and psychosocial interventions improve adaptation [15,44]. Support groups were perceived as valuable spaces for normalizing experiences and reducing isolation [59,60]. In this context, the nurse emerged as the most accessible and trusted professional, consistent with findings from studies on sexual health communication and nursing roles [61]. Regarding the conversational strategy for sensitive topics, an exploratory inductive qualitative study by Juvik et al. found that ostomy patients enrolled in the study recognized the role of specialist nurses and favored structured questionnaires, facilitating discussions on sensitive matters by reducing the awkwardness of direct eye contact and providing a natural starting point for difficult conversations, especially regarding intimate issues [62]. In this study, participants recognized nurses as key figures in detecting distress, initiating conversations about sexuality, and providing tailored education.

### 4.1. Strengths and Limitations

This study presents several strengths and limitations that should be considered when interpreting the findings. A key strength lies in the use of a descriptive phenomenological approach, which allowed for an in-depth exploration of the lived experience of sexuality after stoma formation. The richness of the narratives, supported by the openness of the interview format, offered access to nuanced meanings, emotional processes, and relational dynamics that are often overlooked in quantitative research. The inclusion of participants with different types of stomas and varying clinical trajectories also contributed to capturing a broad range of individual experiences, enhancing the depth and diversity of the data. However, several limitations should be acknowledged. As in all qualitative studies, the subjective and deeply personal nature of the participants’ accounts may limit replicability and restrict the generalizability of the findings to broader populations. Despite the adoption of strategies to enhance methodological rigor, such as phenomenological reduction, triangulation, and analytic supervision, the interpretive process may still be influenced by the researchers’ perspectives. A further limitation concerns the composition of the sample, which included a predominance of men and individuals with a urostomy. While this reflects the epidemiology of the clinical setting from which participants were recruited, it may have shaped the salience of some themes, as sexual experiences can differ across genders and types of stomas. Additionally, the study was conducted within a single hospital in Rome, Italy; this specific setting may limit the transferability of the results to other contexts governed by different social norms, relational expectations, or care models. Overall, while the phenomenological methodology allowed for a rich and meaningful interpretation of participants’ experiences, these limitations should be considered when applying the findings to other populations or contexts.

### 4.2. Implications for Practice

The findings highlight the need to systematically integrate sexual health into the care of individuals living with a stoma. Nurses, often perceived as accessible, trusted, and emotionally attuned professionals, are in a key position to initiate sensitive conversations, identify early signs of emotional or relational distress, and provide tailored education that addresses not only technical stoma management but also intimacy, body image, and partner communication. Embedding structured counselling on sexuality into pre and postoperative care pathways may help normalize the topic, reduce shame or isolation, and support more positive adjustment. Furthermore, implementing targeted training programs for nurses, along with clear referral pathways to professionals in psychology or sexual health, could enhance the quality and completeness of care. Peer support groups may also provide valuable spaces for individuals to share experiences, feel validated, and develop effective coping strategies.

### 4.3. Future Research

Future studies should aim to include more balanced samples in terms of gender and type of stoma, allowing a deeper exploration of potential differences in sexual experiences across subgroups. Longitudinal research would be valuable for understanding how sexuality evolves following stoma surgery, and which factors contribute to resilience, adjustment, or ongoing difficulties. Expanding research to diverse cultural and healthcare contexts could shed further light on the role of cultural norms, taboos, and relational expectations in shaping sexuality after stoma formation. Finally, there is a need to evaluate the effectiveness of structured educational, psychological, or couple-focused interventions, including nurse-led programs, in improving sexual well-being and overall quality of life among individuals living with a stoma.

## 5. Conclusions

Sexuality after stoma formation emerges as an experience profoundly shaped by bodily transformations, emotional vulnerabilities, and relational dynamics. This phenomenological study illuminated the complexity of the lived experiences of individuals with an enterostomy or urostomy, showing how fear of disgust, difficulty recognizing oneself in the altered body, and the partner’s reactions significantly influence intimacy. The findings highlight the importance of systematically integrating sexuality into nursing care and therapeutic education, overcoming the taboos and silences that often characterize the care pathway. Promoting spaces for listening, information, and support may facilitate a more balanced adaptation and improve quality of life, reaffirming sexuality as an essential component of person-centered care.

## Figures and Tables

**Table 1:** Interview guide.

Main Topics	Question
Sexual experience after becoming an ostomate	Could you describe how your experience of sexuality has changed since becoming an ostomate?
Facilitators of sexual well-being	What factors or elements have supported or facilitated your sexual experience?
Barriers to sexual well-being	What factors or elements have hindered or made your sexual experience more difficult?
Suggestions for clinical practice	Do you have any suggestions for how healthcare professionals could better address sexuality in patients with a stoma?

**Table 3:** Example of the coding process in inductive analysis.

Phrases	Categories	Themes
*Our sexuality is almost zero. No, it’s… It’s zero. […] I no longer have erections… My desire has also decreased a lot.”*	Physical distress	**The emotional impact of sexuality after stoma surgery**
*“Since I have the stoma […] I no longer have that energy and desire.”*		
*“My sexuality is dead. […] Now it’s just a memory.”*		
*“My sexual life ended the day before the surgery.”*		
*“Sex has disappeared. Since I had these surgeries, I have a block, a psychological block.”*	Psychological distress	
*“It has dampened me a bit. Mentally I’m positive, but this weighs on me.”*		
*“Before this happened to me, I had a spirit for going with women, whenever I found [one], whenever I saw [one], I had more spirit. Then, since this thing happened to me—it happened that I had my rectum and everything removed—and now I have withdrawn into myself a bit, and I don’t have that drive anymore.”*	Relational distress	
*“There are only some intimate attitudes […], but it’s not like before anymore.”*		
*“You need philosophy. I saved my skin, and that’s what matters.”*	Acceptance and adaptation	

**Table 4:** Prevalence of themes: number of cancer-related quotes per theme and participants.

	The Emotional Impact of Sexuality After Stoma Surgery	The Fear of Disgust and Sexual Rejection	Partner Reactions as a Determinant of Intimacy	Searching for Guidance and Support
**Colostomy**	P4, P5, P15,	P2, P4, P7, P15	P2, P4, P7, P10, P15,	P2, P4, P6, P10, P14
**Ileostomy**	P1, P9, P11, P12, P16	P1, P11, P12	P9, P11, P12	P9, P11, P12, P16
**Urostomy**	P17, P18, P19, P20, P21, P22, P23, P25, P26, P27, P28, P29, P31, P32, P33		P17, P21, P23, P24, P26, P30	P17, P18, P19, P20, P21, P22, P25, P31, P32, P33,

## Data Availability

The data are contained within the article or Supplementary Material.

## Supplementary Materials

The following supporting information can be downloaded at: https://www.mdpi.com/article/10.3390/curroncol33050270/s1, Table S1: COREQ (COnsolidated criteria for REporting Qualitative research) Checklist.

## Abbreviations

The following abbreviations are used in this manuscript:

COREQ	Consolidated Criteria for Reporting Qualitative Research
CECRI	Center of Excellence for Nursing Culture and Research
IFO	Istituti Fisioterapici Ospitalieri
IRCCS	Istituto di Ricovero e Cura a Carattere Scientifico
